# Assessing community awareness, perceptions, ownership, and challenges of stunting reduction programs in Rwanda: a qualitative study

**DOI:** 10.1186/s12889-026-27271-x

**Published:** 2026-04-02

**Authors:** Theobald Mporanyi, Gashaija Absolomon, Felix K. Rubuga, Jeanine Condo, Cyprien Munyanshongore

**Affiliations:** 1 School of Public Health, University of Rwanda, Kigali, Rwanda; 2 Center for Impact, Innovation and Capacity Building in Health Information Systems and Nutrition (CIIC-HIN), Kigali, Rwanda; 3 Tulane School of Public Health and Tropical Medicine, New Orleans, LA USA

**Keywords:** Stunting, Community participation, Nutrition intervention, Qualitative research, Rwanda

## Abstract

**Background:**

Stunting affects 33% of Rwandan children under five despite multiple government interventions. Community engagement critically influences intervention sustainability, yet varying levels of awareness, perception, and ownership persist across districts. This study assessed community awareness, perceptions, and ownership of eight major stunting reduction programs in Rwanda, and identified challenges affecting their success.

**Methods:**

A qualitative assessment design was employed across ten randomly selected districts where stunting reduction programs had been implemented for 5–15 years. Data were collected through 100 Focus Group Discussions (FGDs) with approximately 600 participants, including household heads with children under five, Community Health Workers, and local leaders, and 105 Key Informant Interviews with district officials and program implementers. Data were analyzed thematically, and saturation was reached after analyzing data from eight districts, with two additional districts confirming no new themes emerged.

**Results:**

Community awareness of program objectives was consistently high across all interventions. Participants reported tangible benefits including improved child health, increased immunity, better school attendance, enhanced household income, and greater food security. However, community ownership remained predominantly low, with interventions perceived as government-driven rather than community-led. Key findings are summarized across four themes: high awareness but limited strategic understanding, perceived effectiveness with tangible benefits, weak community ownership and dependency, and systemic challenges including resource constraints, governance issues, and cultural barriers.

**Conclusions:**

While stunting reduction programs demonstrated high awareness and perceived benefits, weak community ownership and systemic challenges threaten sustainability. Strengthening participatory approaches in program design, improving governance transparency, addressing resource constraints, and building local capacity are essential for achieving Rwanda’s stunting reduction targets.

**Clinical trial registration:**

Clinical trial number: not applicable.

## Introduction

Globally, stunting remains one of the most persistent public health challenges, affecting approximately 149 million children under five years of age and reflecting complex interactions between poverty, food insecurity, and inadequate nutrition practices [1]. Stunting, defined as height-for-age below minus two standard deviations from the World Health Organization (WHO) growth standards, has consequences extending beyond physical growth to impair cognitive development, educational attainment, and long-term economic productivity, thereby perpetuating intergenerational cycles of poverty and inequality [2, 3].

In Rwanda, despite substantial economic development and coordinated multi-sectoral efforts, stunting remains a critical concern. The 2019–2020 Rwanda Demographic and Health Survey reported that 33% of children under five were stunted, representing a decline from 38% in 2015 but still reflecting significant chronic undernutrition [4]. This persistent burden has prompted the Government of Rwanda to prioritize stunting reduction within Vision 2050 and the National Strategy for Transformation, with an ambitious target to reduce stunting to 19% by 2024 [5].

To address this challenge, Rwanda has implemented multiple nutrition-sensitive and nutrition-specific interventions, coordinated through the National Food and Nutrition Policy framework and supported by development partners including USAID, UNICEF, WHO, FAO, and bilateral agencies [6, 7]. The major programs examined in this study include:

The Girinka Program (One Cow per Poor Family), launched in 2006, is Rwanda’s flagship livestock transfer program that has distributed over 427,576 cows to poor families across all 30 districts. The program targets households in Ubudehe (social economic categories) categories 1 and 2 (the poorest), aiming to improve household nutrition through daily milk consumption, increase family income through milk sales, and enhance agricultural productivity through manure fertilization. Beneficiaries are selected at the village administrative level (umudugudu) level through community participation, and recipients are expected to pass on the first female calf to another poor family, creating a multiplier effect [8–12].

The One Cup of Milk per Child Program is a school-based nutrition intervention providing daily milk to primary school children to improve attendance, cognitive development, and nutritional status. The program particularly targets schools in districts with high malnutrition rates.

Kitchen Gardens Program launched in early 2010s, this intervention encourages households to establish small vegetable gardens near their homes to improve dietary diversity. The program provides seeds, training on gardening techniques, and nutrition education linking vegetable consumption to child health.

Shisha Kibondo (fortified porridge) also refers to fortified porridge provided to malnourished children and pregnant/lactating women.

The Ongera (Micronutrient Powder) Program distributes micronutrient sachets to children aged 6–23 months through community health workers (CHWs) and health facilities. The powder, added to complementary foods, provides essential vitamins and minerals to prevent and treat micronutrient deficiencies associated with stunting.

The 1,000 Days Campaign focuses on the critical window from pregnancy through a child’s second birthday, promoting exclusive breastfeeding for six months, appropriate complementary feeding, adequate maternal nutrition, and early antenatal care attendance. The campaign is delivered through monthly community work day (Umuganda meetings), CHW home visits, Umugoroba W’ababyeyi (Parents’ Sunset Meeting on nutrition and family well-being) and health facility counseling.

The Ubudehe (social economic categories) Program is Rwanda’s community-based social stratification and social protection system, through which households are classified into socio-economic categories based on their living conditions and capacities, in order to identify vulnerable families and ensure equitable access and promoting transparency and local ownership of beneficiary selection to government support and development programs.

The Crop Intensification Program (CIP) promotes increased agricultural productivity through improved seeds, fertilizers, irrigation, and modern farming techniques, with linkages to nutrition through promotion of bio-fortified crops (orange-fleshed sweet potatoes, high-iron beans) and diversified food production to improve household dietary diversity.

Evidence from multiple contexts demonstrates that nutrition-sensitive agricultural programs can significantly improve dietary diversity and child nutrition outcomes. A meta-analysis by Margolies et al. [13]. Showed that such programs had positive effects on young children’s dietary diversity across low- and middle-income countries. In Rwanda specifically, assessment have shown that the Girinka (One Cow per Poor Family) Program has been associated with improved weight-for-age and height-for-age scores among beneficiary children, with beneficiaries producing an average of 1.5 L more milk daily when properly trained compared to those without training, and manure use contributing to increased crop yields and improved household income [8]. Similarly, kitchen gardening interventions in Pakistan demonstrated improvements in nutrition knowledge and household food practices [14], while Guatemala’s community-based livestock programs showed sustainability and nutritional benefits through local ownership [9].

However, the effectiveness and sustainability of nutrition interventions are strongly influenced by community understanding, acceptance, and active participation [10]. International evidence consistently demonstrates that programs with high community ownership achieve better outcomes and sustainability [11]. In Tanzania, local actors emphasized the importance of sustained political commitment and community engagement in addressing the “triple burden” of malnutrition [12]. Studies in Rwanda have revealed varying levels of community engagement and challenges in program implementation. The Gikuriro (well-growing child) Program assessment found that household ownership of kitchen gardens remained low despite program promotion, with weak enforcement and limited understanding of their nutritional importance [15]. Similarly, while the Girinka (One Cow per Poor Family) Program showed positive nutritional impacts, poverty and food insecurity led some households to prioritize milk sales over consumption, reducing intended benefits for children [16].

Gender dynamics further complicate nutrition outcomes, with traditional norms often limiting women’s decision-making power over household food purchases and consumption despite their primary responsibility for child feeding [17]. A case study by the Japan International Cooperation Agency found inconsistent delivery of nutrition interventions across villages, with caregivers identifying animal-source foods as difficult to obtain due to cost and availability, highlighting gaps between program design and community realities [18].

Despite Rwanda’s comprehensive policy framework and substantial investments in stunting reduction, persistent challenges suggest that understanding community perspectives is essential for improving program effectiveness. While awareness campaigns and service delivery have expanded across the ten intervention districts examined in this study (Nyabihu, Nyamasheke, Nyamagabe, Gisagara, Gakenke, Burera, Gasabo, Kicukiro, Bugesera, and Nyagatare), questions remain about the depth of community understanding of program objectives, perceptions of program effectiveness, levels of genuine ownership and participation in decision-making, and barriers to sustained engagement.

Assessing these dimensions is critical for identifying gaps between program design and community realities, understanding factors influencing sustainability, and informing strategies to strengthen community-based approaches toward achieving national stunting reduction targets.

Therefore, this study aimed to assess community awareness, perceptions, and ownership of stunting reduction programs in Rwanda, and to identify challenges affecting their success. Specifically, the objectives were to: (1) assess community awareness and perceptions regarding the implementation and impact of stunting reduction programs, and (2) investigate the level of community ownership and engagement in the design, implementation, and sustainability of stunting reduction programs.

## Methods

### Study design and setting

This study employed a qualitative design to assess community awareness, perceptions, ownership, and challenges of stunting reduction programs targeting children under five in Rwanda. The qualitative approach enables in-depth understandi ng of social meanings, lived experiences, and contextual factors shaping community engagement and program ownership [19, 20].

The study was conducted in ten randomly selected districts: Nyabihu, Nyamasheke, Nyamagabe, Gisagara, Gakenke, Burera, Gasabo, Kicukiro, Bugesera, and Nyagatare, selected to capture geographical and socio-economic diversity. All ten districts were existing intervention sites where national stunting reduction programs had been implemented for varying durations: Girinka (One Cow per Poor Family) (2006-present, 15 + years), One Cup of Milk per Child (2015-present, 6 + years), Ongera (Micronutrient Powder) (2012-present, 9 + years), Kitchen Gardens (2010-present, 11 + years), 1,000 Days Campaign (2013-present, 8 + years), Ubudehe (social economic categories) (2008-present, 13 + years), and agricultural intensification programs (2008-present, 13 + years). This study was conducted between July and November 2021, representing 5–15 years of program implementation across the various interventions [5, 6].

Data collection for this study was conducted between March and August 2021, representing a cross-sectional assessment of community perspectives after multiple years of program implementation across all districts. Rural districts emphasized agricultural-nutrition linkages, while urban districts focused on social behavior change communication and health facility-based counseling, providing diverse contexts for assessing community engagement with different intervention models.

### Study participants and sampling

Approximately 600 individuals participated in 100 Focus Group Discussions (FGDs), with 5–7 participants per group. Inclusion criteria for household heads were: (1) having at least one child under five years of age, (2) residing in the district for at least one year, (3) being a beneficiary or aware of at least one stunting reduction program, and (4) willingness to participate and provide informed consent. Exclusion criteria included: (1) households with no children under five, (2) temporary residents (less than one year in the district), and (3) those unable to communicate effectively in Kinyarwanda.

Inclusion criteria for Community Health Workers were: (1) active CHW status in the community for at least six months, (2) direct involvement in nutrition-related activities, and (3) willingness to participate. Inclusion criteria for local leaders were: (1) holding leadership position at cell or sector level, (2) involvement in community development programs, and (3) minimum six months in current position. Participants were randomly selected with CHW assistance to ensure diversity in views and experiences. Additionally, 105 Key Informant Interviews (KIIs) were conducted with district officials, health officers, and program representatives using purposive sampling, guided by data saturation principle [21, 22]. Inclusion criteria for KIIs were: (1) official role in planning, implementing, or monitoring stunting reduction programs, (2) minimum one year experience in current position, and (3) willingness to participate.

### Data collection procedures

Semi-structured interview and FGD guides were developed based on study objectives [10, 11], translated into Kinyarwanda, and pilot-tested in Rwamagana District. The data collection team for this study was composed of individuals with substantial educational backgrounds and relevant field experience in collecting data. Enumerators held Bachelor’s degrees in disciplines such as Demography Sciences and Nursing, providing them with a solid foundation in data collection and an understanding of health-related issues. Supervisors possessed Master’s Degrees in Public Health, equipping them with advanced knowledge and skills pertinent to overseeing complex health studies. Prior to commencing fieldwork, all team members, including moderators and note-takers participated in comprehensive training sessions. These sessions covered the study’s objectives, ethical considerations, and the proper use of data collection tools. A significant focus was placed on maintaining neutrality and fostering an open, respectful environment during Focus Group Discussions (FGDs) and Key Informant Interviews (KIIs). The supervisors played a pivotal role in ensuring the integrity of the data collection process. They closely monitored FGD and KII sessions to ensure consistency and completeness, and conducted daily debriefings with data collectors to address any challenges and assess progress. Quality control measures were implemented, including immediate verification of data for completeness and accuracy, as well as random audits of recordings and notes to validate data quality.

FGDs were conducted in accessible community locations (health centers, schools, churches), lasting approximately 60 min. Trained moderators used open-ended questions to explore awareness, perceptions, ownership, and challenges. KIIs were conducted in private settings at interviewees’ workplaces, lasting 45–60 min, covering program design, implementation, and sustainability. All sessions were audio-recorded with explicit consent, and note-takers documented non-verbal cues and group dynamics.

### Data analysis

Data analysis followed a structured approach: decontextualization, recontextualization, categorization, and compilation [23]. FGDs and KIIs were transcribed verbatim in Kinyarwanda and translated into English. Transcriptions were read iteratively to identify recurring patterns, and key meaning units were coded using NVivo software. Codes were reviewed for alignment with study objectives, then grouped into four core themes: (1) awareness of interventions, (2) perceptions of effectiveness and benefits, (3) community ownership, and (4) challenges for success. Data saturation was systematically assessed throughout the analysis process. After analyzing data from the first eight districts, the research team observed no new themes or sub-themes emerging from the transcripts. Analysis of data from the ninth and tenth districts confirmed saturation, as these added only confirmatory examples to already-established themes without introducing new concepts or patterns. The large sample size (705 participants, including 600 participated in FGDs and 105 of KIIs), which were represented by (household heads, CHWs, local leaders, district officials) ensured comprehensive capture of varying perspectives across geographical and socio-economic contexts. Findings were synthesized into a comprehensive narrative, highlighting common themes, variations across districts, and contradictions between different stakeholder perspectives.

### Ethical considerations

The study was conducted in accordance with the principles of the Declaration of Helsinki [24]. Written or verbal informed consent was obtained from all participants, with explicit permission for audio recording. Written consent was obtained from all KII participants and literate FGD participants who could read and sign the consent form. Verbal consent was obtained from FGD participants with limited literacy, which was documented through audio recording and witnessed by a local leader or CHW. This variation in consent procedures was approved by the Institutional Review Board to ensure inclusivity and avoid excluding participants based on literacy levels, which is particularly important in rural areas where literacy rates are lower. Personal identifiers were excluded from transcripts to ensure anonymity. Participation was voluntary, and participants could withdraw without penalty. Tools and procedures respected local customs and languages. The study received ethical clearance from the University of Rwanda, College of Medicine and Health Sciences Institutional Review Board (Approval No. 392/CMHS IRB/2021), ensuring full compliance with national and international research ethics guidelines.

## Results

The following results present qualitative findings from thematic analysis of Focus Group Discussions (FGDs) and Key Informant Interviews (KIIs) conducted across ten districts in Rwanda. Direct quotes illustrate key themes, attributed anonymously through participant identifiers and district locations. The findings reflect community views, opinions, and lived experiences related to eight key interventions aimed at reducing stunting.

### Socio-demographic characteristics of study population

Table 1 presents the socio-demographic characteristics of 705 participants across ten districts. The majority were women (*n* = 550, 78.0%), aged 30–39 years (*n* = 282, 40.0%), from rural areas (*n* = 564, 80.0%), and classified as household heads with children under five (*n* = 530, 75.2%). Educational attainment varied: no formal education (*n* = 127, 18.0%), primary education (*n* = 282, 40.0%), secondary education (*n* = 211, 29.9%), and higher education or technical training (*n* = 85, 12.1%). Socio-economic status based on Ubudehe (social economic categories) classification showed Category 1/poorest (*n* = 212, 30.1%), Category 2/poor (*n* = 282, 40.0%), Category 3/middle (*n* = 176, 25.0%), and Category 4/better off (*n* = 35, 5.0%). The sample included diverse stakeholders: Community Health Workers (*n* = 70, 9.9%), local leaders (*n* = 50, 7.1%), district officials (*n* = 30, 4.3%), health officers (*n* = 15, 2.1%), and program implementers (*n* = 10, 1.4%).

**Table 1:** Socio-demographic Characteristics of Study Participants (*N* = 705)

Variable	Category	Frequency (*n*) / Percentage (%)
Data Collection Method	Focus Group Discussions (FGDs)	600 (85.1%)
	Key Informant Interviews (KIIs)	105 (14.9%)
Gender	Female	550 (78.0%)
	Male	155 (22.0%)
Age Category (years)	20–29	176 (25.0%)
	30–39	282 (40.0%)
	40–49	176 (25.0%)
	50–59	53 (7.5%)
	≥ 60	18 (2.6%)
District (Geographic)	Gasabo	71 (10.1%)
	Kicukiro	70 (9.9%)
	Nyabihu	71 (10.1%)
	Burera	70 (9.9%)
	Gakenke	71 (10.1%)
	Nyamasheke	70 (9.9%)
	Nyamagabe	71 (10.1%)
	Gisagara	70 (9.9%)
	Bugesera	71 (10.1%)
	Nyagatare	70 (9.9%)
Location Type	Rural	564 (80.0%)
	Urban	141 (20.0%)
Educational Level	No formal education	127 (18.0%)
	Primary education	282 (40.0%)
	Secondary education	211 (29.9%)
	Higher education/Technical training	85 (12.1%)
Socio-economic Status	Ubudehe Category 1 (Poorest)	212 (30.1%)
(Ubudehe Classification)	Ubudehe Category 2 (Poor)	282 (40.0%)
	Ubudehe Category 3 (Middle)	176 (25.0%)
	Ubudehe Category 4 (Better off)	35 (5.0%)
Participant Type	Household heads with children < 5 years	530 (75.2%)
	Community Health Workers (CHWs)	70 (9.9%)
	Local leaders	50 (7.1%)
	District officials	30 (4.3%)
	Health officers	15 (2.1%)
	Program implementers	10 (1.4%)

### Overview of core themes and interventions

Each of the eight interventions was systematically assessed based on four core themes. awareness of the program, perceptions of effectiveness and benefits, community ownership, and challenges for success. This structured approach yielded 37 subthemes and 69 subtheme categories, providing a comprehensive understanding of community perspectives across all interventions. The findings demonstrate both commonalities and important distinctions in how different types of interventions were experienced and perceived by communities, as shown in Table 2.

**Table 2:** Summary of Key Findings by Major Themes

Major Theme	Key Findings	Supporting Sub-themes
1. Community Awareness	• High operational awareness • Limited strategic understanding • Gap between activities and purpose	• All 8 programs recognized by communities • CHW outreach and school campaigns effective • **Agricultural programs**: understood inputs but not nutritional links • Confusion about stunting reduction rationale
2. Effectiveness & Benefits	• Positive perceptions across interventions • Tangible multi-domain benefits • Nutrition + poverty programs most valued • Sustainability concerns	**Nutritional**: Improved child health, weight gain, immunity, dietary diversity **Educational**: Better attendance, concentration, reduced dropouts **Economic**: Increased income (milk, manure, yields), food security **Social**: Health insurance access, cooperatives
3. Community Ownership	• Predominantly low ownership • Government-driven perception • Limited decision-making participation • High external dependency	**Girinka** (One Cow per Poor Family): Exclusion from selection, lack of transparency, financial challenges **School Feeding**: Passive parent role, cultural resistance **Supplementation**: Discontinued after free supply ended **Behavior Change**: Gender power imbalances limit women’s autonomy **Top-Down**: Programs driven by imihigo (performance contracts) targets
4. Challenges	• Systemic barriers threaten sustainability • Resource constraints most cited • Governance issues pervasive • Cultural/operational/structural barriers	**Resources**: Water scarcity, limited land (< 300 m²), veterinary access, high costs **Governance**: Favoritism, corruption, weak accountability, misclassification **Cultural**: Food-sharing dilutes impact, milk resistance, gender norms **Operational**: Limited reach, insufficient follow-up, market access issues **Structural**: Short maternity leave, unaffordable foods, climate variability

Synthesizes 37 subthemes across 8 interventions (Girinka (One Cow per Poor Family), One Cup of Milk, Ongera (Micronutrient Powder), Kitchen Gardens, 1,000 Days, Ubudehe (social economic categories), CIP, Food Crops) from 100 FGDs and 105 KIIs in 10 districts

### Community awareness of interventions

Across all ten districts, communities demonstrated consistently high operational awareness of the eight interventions, understanding program activities and immediate benefits. However, this awareness revealed a critical distinction: while participants readily identified what programs offered (milk, seeds, nutritional supplements), many lacked deeper understanding of the strategic linkage between these activities and stunting reduction goals. This gap between operational and strategic awareness was particularly evident in agricultural programs, where communities understood input distribution but struggled to connect these inputs to nutritional outcomes for children under five.

For the Girinka (One Cow per Poor Family) program, participants widely recognized it as a social protection initiative designed to combat stunting and improve household nutrition. Beyond awareness of receiving livestock, beneficiaries articulated concrete health improvements, describing how previously malnourished children transitioned from critical (red) to healthy (green) status on growth monitoring charts, a tangible indicator of the program’s nutritional impact.

*“Girinka is a social protection program aimed at combating stunting. Households that received cows have seen their children recover from malnutrition*,* with health indicators improving from red (critical) to green (healthy)”* (FGD I, Gasabo District).

The One Cup of Milk per Child program was well known among parents and teachers as a school-based intervention aiming to improve child nutrition and reduce stunting. Parents and educators consistently linked the program to multiple benefits: improved attendance, enhanced concentration, and reduced malnutrition among school-aged children, demonstrating awareness that extended beyond milk provision to encompass its broader developmental impacts.

*“We are aware about the program one cup of milk per child at school*,* which helps them stay healthy”* (FGD IV, Nyamagabe District).

Community Health Workers played a key role in raising awareness about the Ongera (Micronutrient Powder) program. Participants recognized it as a government-supported nutritional intervention targeting malnourished children, designed to improve weight and overall child health.

*“Community Health Workers taught us about Ongera Micronutrient Powder Program and how it helps children gain weight”* (FGD II, Gisagara District).

Participants recognized the 1,000 Days Campaign as a vital maternal and child health initiative emphasizing exclusive breastfeeding and appropriate infant feeding practices during the critical first 1,000 days of life. Many recalled receiving information in Umuganda (monthly community work day) meetings and from Community Health Workers.

*“We were told during Umuganda meetings how breastfeeding and eating healthy help children grow well”* (FGD III, Bugesera District).

The Shisha Kibondo (fortified porridge) program was recognized for providing fortified porridge to pregnant breastfeeding mothers and 6-23months old children, with participants understanding its role in maternal and child nutrition during critical periods.

However, while communities understood what programs offered, many lacked understandings of the broader strategic purpose of reducing chronic malnutrition and stunting. This gap was particularly evident in agricultural programs, where communities understood input distribution but not the connection to nutritional outcomes. Some participants associated the Crop Intensification Program only with fertilizers and improved seeds, while others were unsure if it was meant to help them or restrict their farming choices.

*“We were told to grow only maize*,* but no one explained why*,* I don’t know if it is to help us or to control us”* (FGD II, Nyamasheke District).

This disconnect between operational awareness and strategic understanding suggests that while awareness campaigns successfully conveyed program activities, they were less effective at communicating the underlying rationale linking these activities to stunting reduction goals.

### Perceptions of effectiveness and benefits of interventions

Participants across all districts reported tangible benefits from the interventions, demonstrating largely positive perceptions of effectiveness. However, the nature and sustainability of these benefits varied significantly by program type and household capacity. Livestock and supplementation programs delivered immediate, measurable health gains, while agricultural interventions showed more gradual dietary diversification. Economic benefits accrued primarily to households with sufficient resources to maintain program inputs, revealing how structural inequalities mediated program impacts.

### Nutritional improvements

Nutritional improvements emerged as the most consistently reported benefit across all interventions, yet manifestations differed. Girinka (One Cow per Poor Family) beneficiaries described systematic transformations visible through growth monitoring systems, where children progressed from critical to healthy classifications. Ongera (Micronutrient Powder) showed rapid weight gain among previously malnourished children. Shisha Kibondo (fortified porridge) supported maternal and infant health during pregnancy and lactation. Kitchen gardens enabled household-level dietary diversification, particularly adding vegetables to children’s diets.

For the Girinka (One Cow per Poor Family) program, households noted substantial improvements in child nutrition through daily milk consumption. Across all ten districts, participants mentioned the nutritional benefits of milk provided through this program, with the daily availability of milk significantly improving children’s diets. Household members noted stark differences before and after joining the program, observing that previously weak children became much healthier with daily milk consumption.

*“Before receiving a cow*,* my children were weak*,* but now they drink milk every day*,* and they are much healthier”* (FGD III, Gisagara District).

The Ongera (Micronutrient Powder) program was valued for visible health improvements in previously malnourished children. Many parents reported increased weight gain, improved immunity, and general growth among children who had been underweight or malnourished.

*“My child was underweight*,* but after using Ongera*,* his health improved”* (FGD II, Gasabo District).

For the Shisha Kibondo (fortified porridge) program, beneficiaries reported improved maternal and child health due to consumption of fortified porridge. Pregnant women and breastfeeding mothers described how the nutritious porridge ensured good health for both themselves and their babies.

*“I received nutritious porridge during my pregnancy*,* and it ensured good health for both me and my unborn baby”* (FGD I, Gisagara District).

Many households noted that having homegrown vegetables through kitchen gardens improved their diets, allowing them to add more nutritious greens to their meals, especially for children.

*“We now grow vegetables at home*,* which helps us eat healthier meals”* (FGD II, Nyamasheke District).

### Educational benefits

School-based nutrition programs demonstrated a unique dual impact: immediate nutritional supplementation combined with behavioral incentives that improved both attendance and learning outcomes. The One Cup of Milk per Child program created a virtuous cycle where nutritional benefits enhanced cognitive capacity while the prospect of receiving milk motivated regular attendance and punctuality, addressing multiple dimensions of child development simultaneously.

The One Cup of Milk per Child program positively influenced school attendance, punctuality, and learning capacity. The availability of milk at school motivated children to attend more regularly and concentrate better during lessons. Teachers and parents reported that fewer children missed school, with even those who previously came late now arriving on time because they knew they would receive milk.

*“Since the program started*,* fewer children miss school. Even those who used to come late now arrive on time because they know they will get milk”* (FGD III, Nyamasheke District).

Teachers noticed significant differences in children’s energy levels and attentiveness, particularly during afternoon sessions. Children who previously struggled to concentrate by mid-morning now had more energy and could focus better on their lessons.

*“Teachers have noticed a difference. The children are more active and attentive*,* especially in the afternoon sessions*,* compared to before”* (KII I, Bugesera District).

Several participants, especially from rural and low-income backgrounds, noted that the program played a critical role in addressing malnutrition among school-aged children. It was also linked to a reduction in school dropouts, particularly among the most vulnerable children who previously came to school on empty stomachs.

*“Some children used to come to school on an empty stomach*,* but now they at least have something nutritious during the day*,* which helps prevent stunting”* (FGD IV, Burera District).

### Economic and food security benefits

Economic benefits varied substantially by program type and household capacity. While livestock programs created income-generating assets through milk sales and agricultural productivity gains via manure, benefits accrued primarily to households with sufficient resources (land, water, capital) to maintain animals. Agricultural intensification programs showed promise for yield improvements but faced sustainability concerns about soil degradation from repeated fertilizer use and vulnerability to climate variability. Kitchen gardens enhanced household food security by providing vegetables even during scarcity periods, reducing dependence on market purchases.

Economic benefits were emphasized particularly for Girinka (One Cow per Poor Family) and agricultural programs. While all districts recognized the economic value of the livestock program, participants in several districts emphasized this benefit more strongly. Milk sales provided a new revenue stream, and manure was repeatedly cited as a major benefit for farming, with participants reporting improved harvests.

*“The manure we get from cows has improved our farming; we now harvest more”* (KII 1, Nyabihu District).

The integration of kitchen gardens enhanced food security and access to nutritious food at the household level. Households appreciated that having vegetables at home meant they did not always need to buy them, and even when food was scarce, their gardens provided something healthy to eat.

*“Even when food is scarce*,* our gardens give us something healthy to eat”* (FGD VI, Nyagatare District).

For agricultural programs, some farmers reported significant yield improvements, particularly when fertilizers and hybrid seeds were used correctly. Production increases allowed farmers to pay for household expenses including children’s school fees.

*“Using fertilizers doubled my maize harvest compared to what I used to grow”* (FGD II, Bugesera District).

### Social protection benefits

Social protection mechanisms, particularly Ubudehe (social economic category), served as both targeting tools and entry points for integrated support. The system enabled poor households to access Mutuelle de Santé (community-based health insurance) and financial support for income-generating activities. However, effectiveness hinged on classification accuracy and transparent implementation, areas where governance challenges frequently undermined program intent, with reports of misclassification and favoritism limiting benefits to truly vulnerable households.

The Ubudehe (social economic categories) program facilitated access to essential services such as health insurance and education. Many poor households that previously struggled to afford health insurance could now access medical care through Mutuelle de Santé (community-based health insurance).

*“Before Ubudehe*,* many poor households struggled to afford health insurance. Now*,* vulnerable groups can access medical care”* (FGD II, Nyamasheke District).

Community-based support and income-generating activities were strengthened through cooperatives, with households receiving financial support for small businesses that helped improve income and food security.

*“Through Ubudehe*,* we were able to form cooperatives and receive financial support for small businesses”* (FGD IV, Nyamasheke District).

These perceived benefits demonstrate that when interventions address both nutrition and poverty simultaneously, communities recognize them as more effective and impactful. However, the sustainability of these benefits emerged as a concern, particularly for programs reliant on external support.

### Community ownership of interventions

Community ownership emerged as the critical gap undermining program sustainability. Despite widespread appreciation for benefits, ownership remained predominantly low across all interventions, revealing a fundamental paradox: communities valued programs but did not feel they belonged to them. This disconnect manifested differently across intervention types: livestock programs showed sporadic individual success overshadowed by systemic exclusion; nutritional supplementation created dependency rather than autonomy; behavior change campaigns provided information without empowerment; agricultural programs imposed directives without incorporating local knowledge. The root cause was consistent: top-down implementation prioritizing service delivery over participatory design. Programs were perceived as government-driven initiatives meeting performance contract (imihigo) targets rather than community-led solutions addressing locally-defined needs. This perception, combined with limited decision-making power, opaque beneficiary selection, and inadequate capacity building, created an environment where communities viewed themselves as passive recipients rather than active owners.

### Limited ownership in Girinka (one cow per poor family) program

While the majority of participants across the ten districts described limited ownership of the Girinka (One Cow per Poor Family) program, a few respondents did express strong commitment and responsibility for their cows and succeeded in managing them well. Some beneficiaries described a deep sense of responsibility and pride in managing their cows successfully, taking care of them carefully, passing on calves to others, and feeling proud of helping other households.

*“I received my cow five years ago. I took care of it like a child. It gave birth three times*,* and I passed on calves to others without any problem. I feel proud because I have helped other households too”* (FGD III, Nyagatare District).

However, these positive cases were exceptions. The majority described the program as government-led rather than community-driven. Participants consistently described it as a government-led initiative rather than a community-driven effort, with several underlying issues reducing ownership. Being excluded from decisions, especially selection and training, discouraged participants’ sense of responsibility over the program.

*“We applied for a cow*,* but only a few people in our village received them. Some households who needed them most were left out without clear explanations. Because of this*,* we feel left out and disconnected from the program”* (FGD II, Nyagatare District).

Participants expressed that lack of transparency in the selection process should involve the community more, as unclear criteria prevented them from feeling ownership.

*“Leaders choose who gets the cows*,* but we don’t understand their selection criteria. It should be more transparent and involve the community. If we are not part of the process*,* we do not feel ownership”* (FGD IV, Nyamagabe District).

Participants explained that financial challenges made it difficult to sustain the cows, weakening their sense of ownership. The expense of feeding cows forced some beneficiaries to sell them, and when they could not afford to sustain what they received, it no longer felt like it belonged to them.

*“I received a cow*,* but feeding it is expensive. I had to sell it because I could not afford to keep it. When we can’t afford to sustain what we receive*,* it no longer feels like it belongs to us”* (FGD I, Rwamagana District).

Participants emphasized that exclusion from cooperatives left them unsupported, reducing their sense of belonging to the program. If households could organize themselves in cooperatives to share veterinary services and buy animal feed together, it would be more affordable and increase their sense of participation.

*“If we organized ourselves in cooperatives*,* we could share veterinary services and buy animal feed together*,* making it more affordable. Without this*,* each household struggles alone*,* and we don’t feel we are part of cow program”* (FGD IV, Nyagatare District).

### Passive ownership in school feeding program

For the One Cup of Milk program, despite widespread appreciation, responses from participants clearly revealed a significant lack of community ownership. Most viewed the initiative as entirely government-driven, with minimal involvement from parents or local stakeholders. Many participants highlighted that parents play a passive role, with some even undermining the program by discouraging their children from consuming milk due to cultural beliefs.

*“Some parents tell their children not to drink the milk at school*,* because of cultural beliefs”* (FGD II, Bugesera District).

Participants noted that exclusion of local farmers and cooperatives from the supply chain further weakened community ownership. If local farmers supplied the milk, communities would feel more connected to the program and support it more actively.

*“If local farmers supplied the milk*,* we would feel more connected to the program and support it more”* (FGD IV, Nyamagabe District).

### Dependency in Ongera (micronutrient powder) programs

Participants clearly indicated that ownership of the micronutrient supplementation program remained weak, with many households discontinuing use once free distributions ended. Communities viewed the program as external aid rather than a practice they should sustain themselves.

*“When we receive Ongera*,* we use it*,* but when it is no longer given*,* we stop. We cannot afford to buy it ourselves”* (FGD IV, Nyamasheke District).

In multiple FGDs, participants admitted they lacked knowledge and skills on how to maintain good nutrition without relying on micronutrient supplements. While they were told about the importance of micronutrients, they were not trained on how to replicate the nutritional value using locally available ingredients.

*“They told us children need micronutrients*,* but they never trained us on how to get them without Ongera”* (FGD III, Gisagara District).

Similarly, kitchen gardens declined after initial support ended. Despite initial enthusiasm, fewer households-maintained kitchen gardens due to lack of sustained support and resources.

*“Most people stopped when the trainings and follow-up ended”* (FGD III, Burera District).

### Limited ownership in behavior change programs

For the 1,000 Days Campaign, while mothers actively participated in community discussions, health club meetings, and support groups, their involvement often remained informational rather than empowering. Without the means or enabling environment to implement what they learned, ownership was limited. Women met in groups to discuss Infant and Young Child Feeding (IYCF) practices and breastfeeding, but sometimes felt they were just talking without a real way to apply knowledge in their daily lives.

*“We meet in women’s groups to discuss what we learn about IYCF practice and breastfeeding*,* but sometimes we feel like we are just talking without a real way to apply it in our daily lives”* (FGD II, Gasabo District).

Gender roles and power imbalances in households limited mothers’ autonomy over nutrition decisions. Husbands controlled household money, preventing mothers from purchasing needed foods even when they knew what their children should eat.

*“Our husbands control the money*,* so sometimes we can’t buy the food we need. When we ask for money to buy meat or fish*,* they say it’s a luxury*,* and we should just eat beans”* (FGD I, Kicukiro District).

### Top-down implementation of social protection and agricultural programs

Many participants felt that the Ubudehe (social economic categories) program was owned more by the government than by the community. The top-down nature of decision-making, where communities were not involved in project design, household selection, or allocation of support, created a perception that the initiative was externally imposed.

*“The program is meant to be for the community*,* but sometimes leaders decide without involving us”* (FGD III, Nyabihu District).

For agricultural programs, although farmers actively participated, their limited control over key decisions significantly weakened their sense of ownership. Government directives on crop selection and farming techniques imposed rigid guidelines, leaving little room for households to incorporate traditional knowledge and preferences.

*“We are told what to grow*,* but we wish we had more say in choosing crops that fit our land and household needs”* (FGD III, Nyamagabe District).

Across all interventions, participants consistently expressed that programs were implemented to meet local leaders’ imihigo (performance contract) targets. This led to short-term, quota-driven interventions rather than long-term, holistic solutions.

*“Some programs are implemented to meet local leaders’ imihigo targets*,* for example*,* reducing the number of children in ‘red’ malnutrition categories*,* leading to short-term interventions rather than long-term solutions”* (FGD I, Gasabo District).

This perception of externally imposed programs, combined with limited decision-making power and insufficient capacity building, created an environment where communities viewed themselves as passive recipients rather than active owners of stunting reduction efforts.

### Challenges for success

Five challenge categories emerged—resource constraints, governance issues, cultural barriers, operational inefficiencies, and structural limitations—but analysis revealed these were not discrete problems. Rather, they formed an interconnected system of barriers reinforcing each other. Resource constraints (water scarcity, land limitations, veterinary access) were exacerbated by governance failures (favoritism in selection, weak accountability), which in turn undermined community ownership. Cultural barriers (gender power dynamics, food-sharing practices) intersected with structural limitations (short maternity leave, unaffordable nutritious foods) to constrain women’s autonomy over nutrition decisions. Operational challenges (limited program reach, insufficient follow-up) were amplified by policy gaps and environmental vulnerabilities.

### Resource constraints

Resource limitations emerged as the most frequently cited challenge, affecting multiple interventions simultaneously. Water scarcity undermined livestock programs, land constraints prevented fodder cultivation and kitchen garden maintenance, veterinary service gaps left animals untreated, and financial burdens made program benefits inaccessible to the poorest households, the very populations programs aimed to support.

Water scarcity was particularly problematic for livestock and agricultural programs, especially in drought-prone areas affecting both livestock and household needs. During droughts, finding water for cows became impossible, with households struggling to access clean water even for themselves.

*“During droughts*,* finding water for the cows becomes impossible. Even households struggle to access clean water for themselves. How can we keep the cows healthy?”* (FGD V, Burera District).

Participants noted that the government did not consider water availability when distributing cows. In some areas, households had to walk kilometers to fetch water just to keep animals alive, and lack of water affected milk production.

*“When it doesn’t rain for a long time*,* our cows stop producing milk because they don’t drink enough water. The program should support boreholes for cattle”* (FGD III, Nyagatare District).

Land constraints were reported particularly by households living in Umudugudu (village administrative level) settlements with limited plot sizes. Participants described living in settlements where plots were less than 300 square meters, making it infeasible to grow fodder for cows and forcing them to buy expensive feed.

*“We live in Umudugudu settlements where plots are less than 300 square meters. Growing fodder for cows is not feasible*,* so we have to buy feed*,* which is very expensive”* (FGD II, Gakenke District).

Veterinary services and maintenance costs posed additional challenges. Households reported difficulties affording treatment and accessing veterinarians, particularly in remote areas where veterinarians were far away and help often arrived too late.

*“In remote areas*,* veterinarians are far away. When the cow gets sick*,* we have to walk long distances*,* and by the time help arrives*,* it may be too late”* (FGD III, Nyabihu District).

The financial burden of maintaining cows was another major challenge. Feeding, medication, and shelter requirements made cows a liability rather than an asset for those without sufficient funds.

*“Cows need regular feeding*,* medication*,* and shelter. For some of us*,* this becomes a burden rather than a benefit”* (FGD II, Gasabo District).

### Governance issues

Governance challenges permeated all interventions, manifesting as favoritism in beneficiary selection, corruption in implementation, lack of transparency in criteria, and weak accountability systems. These failures directly undermined community ownership by reinforcing perceptions that programs served political or personal interests rather than community needs. When selection processes lacked transparency and accountability mechanisms were weak, even well-designed programs failed to build trust or achieve equitable outcomes.

Participants consistently highlighted concerns that not all eligible households received support, with favoritism being common and some financially stable people receiving benefits while the poorest were left out.

*“Not all eligible households receive cows; favoritism is common. Some people who already have financial means are given cows*,* while the poorest among us are left out”* (FGD II, Nyagatare District).

Some participants raised concerns that cows were given to relatives of leaders without community consultation, with processes manipulated to benefit those close to local authorities.

*“In our sector*,* some cows were given to leaders’ relatives. When we asked why*,* they told us they were following government lists*,* but we were never consulted”* (FGD IV, Nyabihu District).

Lack of transparency in selection criteria created confusion and resentment. Communities did not understand how beneficiaries were selected, with some eligible households never receiving support while others less in need were given priority.

*“We don’t understand how beneficiaries are selected. Some households that meet all the requirements never receive cows*,* while others who are less in need are given priority”* (FGD II, Gakenke District).

Weak accountability systems undermined program effectiveness, particularly in the calf pass-on system. Participants revealed that lack of accountability undermined shared responsibility and community trust.

*“The follow-up on whether people have passed on the calves is weak. Some beneficiaries keep all the cows*,* and others never get one. Without follow-up*,* we feel the program is not fair or managed well”* (FGD I, Nyamagabe District).

For the Ubudehe (social economic categories) program, misclassification of households resulted in unfair distribution of support, with vulnerable households excluded while better-off families were sometimes included.

*“Some people who are financially stable are classified as poor*,* while truly needy households are left out”* (FGD II, Gasabo District).

### Cultural barriers

Cultural factors reduced program impact through multiple pathways. Food-sharing practices diluted benefits intended for specific beneficiaries. Religious beliefs and traditional norms discouraged consumption of certain foods. Gender power dynamics limited women’s decision-making authority over household nutrition despite their primary caregiving responsibilities. These cultural barriers were not merely individual preferences but structural constraints embedded in household and community relations, requiring interventions that addressed power dynamics rather than simply providing information or resources.

Food-sharing practices diluted the benefits of targeted nutritional support. For example, Shisha Kibondo (fortified porridge) assistance provided only to stunted children resulted in food being shared with other family members or sold to meet broader household needs.

*“Shisha Kibondo assistance is provided only to stunted children*,* which can result in the food being shared with other family members or sold to meet broader household needs”* (FGD II, Burera District).

Cultural resistance to milk consumption undermined the school feeding program. In some households, cultural beliefs or household economic needs reduced the program’s intended impact, with parents discouraging their children from consuming milk at school.

*“Some parents tell their children not to drink the milk at school*,* either because of cultural beliefs (abayohova religious) or because they want to share it with household members”* (FGD I, Kicukiro District).

Gender power dynamics limited women’s autonomy over nutrition decisions. Traditional norms limited women’s decision-making power over household food purchases and consumption despite their primary responsibility for child feeding.

*“Our husbands control the money*,* so sometimes we can’t buy the food we need”* (FGD I, Kicukiro District).

### Operational barriers

Operational challenges revealed systemic weaknesses in program design and delivery. Limited reach left vulnerable populations without access. Insufficient follow-up support allowed households to revert to previous practices once external assistance ended. Market access constraints prevented households from realizing economic benefits. Distribution delays, supply chain inefficiencies, and logistical obstacles created implementation gaps. These operational failures were compounded by structural barriers: inadequate maternity leave preventing exclusive breastfeeding, unaffordable nutritious foods creating gaps between knowledge and practice, environmental vulnerabilities exposing programs to climate risks, and policy frameworks prioritizing service delivery over sustainable capacity building.

Limited program reach left many vulnerable households without access. The program’s limited reach was highlighted as a major barrier, with not all children in need able to access services.

*“The program doesn’t reach every household*,* and many children in certain areas are left out”* (FGD III, Burera District).

Insufficient follow-up support after initial program implementation led to declining participation. Participants reported that once support from the health system ceased, many households reverted to previous dietary patterns.

*“After receiving Ongera*,* they leave us on our own*,* and we slowly go back to what we used to do”* (FGD IV, Gisagara District).

Market access challenges limited economic benefits of livestock and agricultural programs. Participants explained that limited market access for milk made cows less beneficial and weakened motivation to engage.

*“Milk prices are too low to generate meaningful income. People in our community cannot afford to buy milk*,* so we have no market”* (FGD I, Nyagatare District).

For agricultural programs, distribution delays, affordability issues, and supply chain inefficiencies made it difficult for some participants to benefit fully.

*“By the time we receive fertilizers*,* the planting season is almost over”* (FGD I, Nyagatare District).

Logistical challenges such as poor road access and limited storage facilities created obstacles for consistent supply and delivery.

*“In rainy seasons*,* the roads are impassable*,* and we do not receive the supplies on time”* (FGD II, Nyagatare District).

### Structural and policy barriers

Structural constraints limited the ability of mothers to adopt recommended behaviors. Working mothers faced difficulties practicing exclusive breastfeeding due to short maternity leave periods.

*“Maternity leave is too short*,* and it makes it hard for working mothers to breastfeed exclusively”* (KII 4, Gasabo District).

Despite awareness, many households struggled to afford recommended nutritious foods, which hindered implementation of infant and young child feeding guidelines. Financial barriers created a gap between knowledge and practice, limiting effective ownership.

*“They advise us to eat certain foods*,* but these foods are too expensive”* (FGD II, Burera District).

For agricultural programs, environmental sustainability concerns emerged. The program’s reliance on rain-fed agriculture increased vulnerability to climate variability, and repeated fertilizer use raised concerns about long-term soil health.

*“Using fertilizers every season is damaging our soil quality”* (FGD III, Burera District).

These interconnected challenges reveal that while individual interventions showed promise, their effectiveness was constrained by systemic barriers requiring comprehensive, multi-sectoral approaches. Addressing stunting reduction demands not only program improvements but fundamental shifts in governance transparency, gender equity, resource allocation, and community empowerment, moving from service delivery to capacity building, from top-down directives to participatory design, from creating beneficiaries to empowering actors. Without addressing these underlying structural and systemic issues, even well-intentioned programs risk perpetuating dependency rather than fostering sustainable, community-owned solutions.

## Discussion

This study assessed community awareness, perceptions, ownership, and challenges of stunting reduction programs in Rwanda. The findings reveal a complex landscape where high operational awareness and perceived benefits coexist with weak community ownership and substantial implementation barriers, highlighting critical gaps between program design and community realities. The assessment demonstrates that while Rwanda’s multi-sectoral approach has achieved important gains in awareness and short-term benefits, fundamental weaknesses in participatory implementation, governance transparency, and capacity building threaten long-term sustainability and impact.

### Community awareness and understanding of interventions

Community awareness of program objectives was consistently high across all interventions, with participants clearly identifying main objectives related to improving child health and nutrition. This echoes evidence from Tanzania, where researchers reported change that social behavior changes communication and school feeding programs enhanced knowledge and visibility of nutrition interventions [25]. Similarly, in Pakistan, researchers found that kitchen gardening interventions significantly raised awareness and improved household nutrition practices [14].

However, while communities in Rwanda knew what programs offered, many lacked understandings of the broader objectives of reducing chronic malnutrition and stunting. This gap was particularly evident in agricultural programs, where communities understood distribution of inputs but not the connection to nutritional outcomes. This aligns with findings from the Gikuriro (well-growing child) Program evaluation, which showed that kitchen garden ownership in Rwanda was low partly because beneficiaries were not adequately informed of long-term nutritional goals [15]. Thus, while awareness of program activities was high, comprehension of their strategic purpose remained limited, suggesting the need for more comprehensive nutrition education that explicitly links agricultural and nutritional interventions to child growth outcomes.

### Perceived effectiveness and benefits of interventions

Perceptions of intervention effectiveness were largely positive, with communities reporting tangible gains including improved child health, weight gain, immunity, better school attendance, enhanced maternal nutrition, and increased household food security and income. For Girinka (One Cow per Poor Family), beneficiaries highlighted daily milk consumption and manure for crop productivity, consistent with Flax et al. who found improved weight-for-age and height-for-age scores among program households and documented challenges when poverty forced families to prioritize milk sales over consumption [8, 16].

The One Cup of Milk program was associated with better school attendance, reduced dropouts, and improved concentration, reflecting evidence from school feeding programs globally [13]. A systematic review and meta-analysis by Margolies et al. demonstrated that nutrition-sensitive agricultural programs have significant positive effects on young children’s dietary diversity across low- and middle-income countries [13]. Ongera (Micronutrient Powder) was valued for improving child weight and immunity, though its sustainability was questioned once free supplies ended, highlighting dependency issues common to supplementation programs [26].

The dual benefit of nutrition and income generation was particularly appreciated in programs like Girinka (One Cow per Poor Family) and kitchen gardens, reinforcing evidence from Guatemala’s Rabbit Project, where women valued both protein supply and income [9]. Similarly, in Pakistan, kitchen gardening improved both household diets and economic resilience [14].These findings confirm that when interventions address both nutrition and poverty simultaneously, communities perceive them as more effective and impactful, supporting the importance of nutrition-sensitive approaches recommended by the Food and Agriculture Organization [24].

### Low community ownership and dependency on external support

Despite widespread appreciation, community ownership of stunting interventions in Rwanda was generally low. Most programs were perceived as government- or donor-driven, with limited involvement of communities in design, planning, and monitoring. Beneficiaries often felt excluded from decision-making, as reported in Girinka (One Cow per Poor Family) selection processes, or disconnected from supply chains, as seen in the One Cup of Milk program where milk was sourced centrally rather than locally. Similar patterns were found in Ongera (micronutrient powder) and Shisha Kibondo (fortified porridge), where reliance on external support led to dependency and limited long-term adoption.

This finding reflects broader challenges highlighted in Rwanda’s Gikuriro (well-growing child) program evaluation, which showed that weak household ownership of kitchen gardens reduced sustainability [15]. It also mirrors evidence from Tanzania, emphasized that interventions achieved results but struggled with long-term community engagement [25]. Globally, literature shows that interventions with high ownership, such as Guatemala’s Rabbit Project where women actively managed livestock, are more sustainable [9]. A systematic review by Tumilowicz et al. emphasized that community ownership and participation are critical determinants of sustained behavior change in nutrition programs [11].

Rwanda’s programs risk dependency and decline if ownership is not strengthened through meaningful community participation in all program phases. The perception that programs were implemented to meet local leaders’ imihigo (performance contract) targets rather than being co-created with communities further undermined trust and sustainability. This top-down approach contradicts principles of community-based participatory research and raises questions about the long-term viability of current implementation strategies [27].

### Implementation challenges: resource, governance, and cultural barriers

Participants identified significant challenges undermining intervention success, including resource constraints (lack of land, water, veterinary services, agricultural inputs, and storage facilities), governance issues (favoritism and corruption in beneficiary selection), and operational barriers (irregular supplies, poor follow-up, and weak accountability in calf pass-on systems). Cultural barriers also limited effectiveness, such as parental resistance to school milk consumption or food-sharing practices that diluted the impact of targeted support.

These challenges resonate with findings across sub-Saharan Africa. Resource limitations and cultural practices hindered sustained progress in similar contexts [25, 28]. In Rwanda, despite Girinka’s (One Cow per Poor Family) successes, poverty and food insecurity forced families to prioritize milk sales over child consumption, reducing program impact [16]. A case study by the Japan International Cooperation Agency18 highlighted that while growth monitoring was common, other interventions like cooking demonstrations were inconsistently delivered, reducing long-term gains, mirroring our findings on inadequate follow-up support.

Gender dynamics emerged as a critical barrier, with traditional norms limiting women’s decision-making power over household food purchases despite their primary responsibility for child feeding. This aligns with Farnworth et al. who documented how male-dominated decision-making processes and inadequate financial support from men reduced household consumption of nutritious foods in Rwanda [17]. Addressing these gender-based constraints requires engaging men in nutrition education and promoting shared household decision-making, as recommended by recent gender-transformative nutrition interventions [29].

Importantly, many interventions were perceived as “imposed from above,” linked to imihigo (performance contracts) and political priorities, rather than being co-created with communities. This top-down perception weakened trust, reduced equity, and limited long-term sustainability. Evidence from community-based participatory interventions demonstrates that co-design approaches lead to greater ownership, cultural appropriateness, and sustained behavior change [27, 30].

Governance challenges, particularly favoritism and corruption in beneficiary selection, emerged as pervasive issues across programs. Misclassification in Ubudehe (social economic categories), opaque selection criteria for Girinka (One Cow per Poor Family), and weak accountability mechanisms undermined program credibility and community trust. These findings align with broader evidence on the importance of transparent, equitable targeting mechanisms in social protection programs [31]. Strengthening community oversight through participatory monitoring mechanisms, such as community scorecards, could enhance accountability and reduce corruption [32].

### Strengths and limitations

This study benefited from several methodological strengths. The multi-sectoral, multi-district sampling approach engaging 705 participants across 100 FGDs and 105 KIIs enabled comprehensive exploration of diverse perspectives from household beneficiaries, community health workers, local leaders, and program implementers. The systematic thematic analysis across eight major interventions provided robust evidence on both common patterns and intervention-specific challenges. The use of both FGDs and KIIs allowed triangulation of community and stakeholder perspectives, enhancing credibility of findings.

Nonetheless, the study has limitations that must be acknowledged. First, the focus on ten districts, while geographically diverse, may not capture experiences from all 30 districts in Rwanda, potentially limiting generalizability. Second, the study relied on self-reported perceptions rather than objective outcome measurements, which may be subject to recall bias or social desirability bias. Third, while data saturation was achieved, the cross-sectional design limits our ability to assess how perceptions and ownership evolve over time. Longitudinal studies tracking community engagement and program sustainability would strengthen understanding of these dynamics. Finally, the study did not systematically assess the differential impact of interventions on vulnerable subgroups, particularly examining how gender, poverty level, and geographic location intersect to shape program experiences and outcomes.

### Policy and programmatic implications

The findings have important implications for policy and practice. First, Rwanda should prioritize strengthening community participation mechanisms throughout the program cycle, from design and targeting to monitoring and evaluation. Establishing mandatory requirements for community engagement, as recommended by Gillespie et al. would shift programs from top-down directives toward co-created solutions [10]. Second, governance mechanisms must be strengthened to reduce favoritism, improve transparency in beneficiary selection, and enhance accountability through community scorecards and participatory monitoring systems [32].

Third, addressing structural constraints such as inadequate land, water systems, and veterinary services requires increased investment in infrastructure that community-level actors cannot fix alone. Fourth, sustainability planning should be integrated from program inception, with clear strategies for transitioning from external support to community-owned solutions. This includes capacity building on locally sustainable nutrition practices, such as producing fortified foods from local ingredients rather than continued dependency on supplements like Ongera (Micronutrient Powder).

Finally, programs must explicitly address gender dynamics and household power imbalances that constrain women’s ability to implement recommended nutrition practices. Gender-transformative approaches that engage men as partners in child nutrition, as demonstrated in other contexts, should be systematically integrated into Rwanda’s stunting reduction programs [29].

Sixth, nutrition education must evolve from activity-focused messaging to comprehensive understanding of the causal pathways linking interventions to stunting reduction. Communities need to understand not only what to do (grow vegetables, feed children milk) but why these actions matter for child growth and development. Seventh, program implementation should shift from meeting imihigo (performance contract) targets to fostering genuine behavior change and community capacity. This requires revising performance metrics to include ownership indicators such as community participation rates, local resource mobilization, and sustained adoption beyond program support.

### Future directions

Future research should pursue several critical directions to strengthen evidence and inform policy. First, longitudinal studies tracking program participants over 3–5 years would illuminate sustainability trajectories and identify factors predicting successful community ownership transitions. Such studies should explicitly examine how ownership evolves as external support diminishes and which program features foster lasting behavior change.

Second, comparative implementation research across Rwanda’s 30 districts would identify effective local adaptations and contextual factors enabling success despite systemic challenges. This research should document innovative governance models, community mobilization strategies, and resource-sharing mechanisms that achieve equitable outcomes. Rigorous evaluation of participatory targeting approaches versus administrative selection could inform optimal beneficiary identification systems.

Third, intersectional analyses examining how gender, poverty, geography, and household structure interact to shape program access, participation, and outcomes would enable more equitable design. Research should specifically investigate barriers facing female-headed households, landless families, and communities in drought-prone areas. Understanding these intersections can guide targeted strategies addressing multiple vulnerabilities simultaneously.

Fourth, cost-effectiveness analyses comparing top-down versus participatory implementation models would inform resource allocation decisions. While participatory approaches require greater upfront investment in community mobilization and capacity building, they may prove more cost-effective long-term if they reduce dependency and enhance sustainability. Such analyses should account for both direct program costs and broader economic impacts on household income and child productivity.

Fifth, implementation science research testing specific strategies to strengthen ownership would generate actionable evidence. Potential interventions include: community-led targeting using participatory wealth ranking; local procurement systems connecting farmers to school feeding programs; cooperative models pooling resources for shared challenges like veterinary care; and participatory monitoring mechanisms enabling community oversight. Rigorous evaluation using cluster-randomized designs would identify which approaches most effectively build sustainable ownership.

Sixth, research examining the political economy of stunting reduction programs would illuminate how imihigo (performance contract) systems, resource flows, and accountability structures shape implementation. Understanding these dynamics is essential for designing governance reforms that align political incentives with community empowerment rather than short-term, quota-driven results.

Finally, given the interconnected nature of identified challenges, systems thinking approaches and complexity-informed evaluation would better capture how interventions interact with existing health, agricultural, and governance systems. Such research would move beyond linear cause-effect models to understand feedback loops, unintended consequences, and emergent properties shaping program outcomes. This systems perspective is essential for designing integrated, adaptive solutions addressing stunting’s multifaceted determinants.

## Conclusion

Rwanda’s stunting reduction programs have achieved important gains in awareness and generated valued benefits, but weak community ownership threatens sustainability. The paradox of high awareness coupled with low ownership stems not from technical program deficiencies but from fundamental governance and design failures: top-down implementation excluding community voice, opaque targeting undermining trust, insufficient capacity-building creating dependency, and structural constraints limiting women’s agency. Addressing these challenges requires more than programmatic adjustments. It demands a paradigm shift from delivery to facilitation, from beneficiary selection to community co-creation, from coverage targets to ownership indicators. Only when communities become genuine agents shaping interventions can Rwanda’s impressive short-term gains translate into sustained stunting reduction. The path forward is clear: Rwanda must choose between continuing to deliver benefits that communities cannot maintain or empowering communities to own solutions they will sustain.

## Data Availability

The qualitative data (FGD and KII transcripts) analyzed during this study contain sensitive information that could compromise participant confidentiality and are therefore not publicly available. De-identified data supporting the findings are available from the corresponding author upon reasonable request and subject to ethical approval for secondary use. Data requests should specify the research purpose and demonstrate appropriate ethical oversight.
